# Population scale retrospective analysis reveals distinctive antidepressant and anxiolytic effects of diclofenac, ketoprofen and naproxen in patients with pain

**DOI:** 10.1371/journal.pone.0195521

**Published:** 2018-04-18

**Authors:** Tigran Makunts, Isaac V. Cohen, Kelly C. Lee, Ruben Abagyan

**Affiliations:** Skaggs School of Pharmacy and Pharmaceutical Sciences, University of California San Diego, La Jolla, California, United States of America; Chiba Daigaku, JAPAN

## Abstract

Currently approved monoamine modulating antidepressant and anxiolytic pharmaceutics fail in over one third of patients due to delayed and variable therapeutic effect, adverse reactions preceding the therapeutic action, and adherence issues. Even with adequate adherence to the regimen and tolerability, one third of the patients do not respond to any class of antidepressants. There is a strong correlation between treatment resistant depression and increase in inflammatory cytokines in plasma and cerebrospinal fluid. Furthermore, epidemiological studies suggest that depression and anxiety are commonly comorbid with pain and inflammation. While a link between pain, inflammation and depression has been suggested it remains unclear which anti-inflammatory treatment may be beneficial to patients with depression and anxiety due to pain. Here, we analyzed 430,783 FDA adverse effect reports of patients treated for pain to identify potential antidepressant and anxiolytic effects of various anti-inflammatory medications. Patients treated for depression or patients taking any known antidepressants were excluded. The odds ratio analysis of 139,072 NSAID reports revealed that ketoprofen was associated with decreased reports of depression by a factor of 2.32 (OR 0.43 and 95% Confidence Interval [0.31, 0.59]) and decreased reports of anxiety by a factor of 2.86 (OR 0.35 [0.22, 0.56]), diclofenac with decreased depression reports by a factor of 2.22 (OR 0.45 [0.40, 0.49]) and anxiety by a factor of 2.13 (OR 0.47 [0.41, 0.54]), while naproxen decreased depression reports by a factor of 1.92 (OR 0.52 [0.49, 0.57]) and anxiety by a factor of 1.23 (OR 0.81 [0.75, 0.88]). Other NSAIDs did not exhibit any noticeable antidepressant and/or anxiolytic effect.

## Introduction

Depressive disorders represent the third leading contribution to the global disease burden. In majority of the industrialized countries, the lifetime prevalence of depression ranges between 8–12%[1, 2].

The standard of treatment for depression consists of 5 main classes of antidepressants, all of which act on monoamine neurotransmitter pathways. Nearly half of the patients who take antidepressants discontinue therapy prematurely due to late onset of beneficial effects, adverse effects, and fear of dependence[3, 4]. The beneficial effect of antidepressants may not be observed for 2–3 weeks, and the maximum effect can take up to eight weeks of therapy[4]. According to a STAR*D study, with adequate adherence and tolerability, the remission rate for depression was estimated to be over 50% after two therapeutic trials of antidepressants including a selective serotonin reuptake inhibitor (SSRI), a dopamine-norepinephrine reuptake inhibitor (DNRI), and a serotonin-norepinephrine reuptake inhibitor (SNRI). The remission rate increased to 67% after therapeutic trials of four antidepressant regimens were used. Unfortunately, this leaves one third of the patients who fall into the treatment resistant depression (TRD) category[5]. Many clinicians turn to off-label uses of other medications as primary or adjunct treatment for depression, bipolar depression, and TRD[6–11]. These include ketamine, minocycline and NSAIDs such as celecoxib, aspirin and diclofenac.

In an earlier study, we performed a statistical analysis of reports from the FDA Adverse Event Reporting System (FAERS) and saw a significant decrease of depression rates in patients who received ketamine, minocycline, botulinum toxin, and diclofenac, when compared to patients prescribed other pain medications[12]. Rates of depression with diclofenac were particularly interesting, but its efficiency and relative antidepressant and anxiolytic efficacy in comparison with other NSAIDs remained unclear.

Diclofenac was developed in 1973 as an analgesic agent, and since then has been commonly prescribed worldwide[13]. It preferentially inhibits cyclooxygenase (COX)-2 resulting in antipyretic, analgesic, and anti-inflammatory effects through decreased prostaglandin E2 (PGE2) levels. Similar to other NSAIDs, diclofenac has dose-dependent renal, gastrointestinal, and cardiovascular toxicities[14, 15]. In addition to our previous FAERS analysis findings[12], diclofenac was studied for its antidepressant effect in comparison to ketamine in a trial with 40 chronic pain patients[8]. It was also discovered that in rat models, diclofenac restored interferon (INF)-alpha induced increase in monoamine neurotransmitter turnover[16]. The link between pain, inflammation and mood disorders has been studied extensively due to increased levels of cytokines and prostaglandins among patients with major depressive disorders and TRD[17–19]. Subsequently, investigators have found that NSAIDs including celecoxib[7, 10], diclofenac [8], and aspirin[20, 21], may have antidepressant effects in pain and inflammation patients and this effect has been generally attributed to COX1/2 inhibitions[9, 22].

While numerous evidence suggested links between pain, inflammatory response, and depression[23–26], it remained unclear if all NSAIDs would have an antidepressant effect. Here, we analyzed over four hundred and thirty thousand FAERS reports of patients treated for pain to compare and contrast the antidepressant and anxiolytic effects of different NSAIDs including diclofenac. The results revealed unique effects of ketoprofen, diclofenac, and naproxen that may be attributable to their specific additional pharmacology rather than their COX-1/2 mediated anti-inflammatory effects (S2 Fig and S5 Appendix).

## Methods

The study used over nine million reports available from the FDA FAERS and AERS data sets. At the time of the study, the FAERS set contained data from September 2012 to March 2017, and its older AERS set contained data from January 2004 to August 2012. The reports were used to run a retrospective data analysis on the drugs of interest. Both FAERS and AERS data sets are available online at: http://www.fda.gov/Drugs/GuidanceComplianceRegulatoryInformation/Surveillance/AdverseDrugEffects/ucm082193.htm.

### Combining and normalizing the data

The quarterly online reports are not homogeneous from year to year and from quarter to quarter throughout the data set. For our study it was necessary to individually download each quarterly data set in dollar separated text (.TXT) format and modify text tables to produce a consistent table field structure. The column names were homogenized and missing columns in older data sets were added with no values. Reports were organized into single primary I.D. tables. The final version of the data set contained over 9.2 million individual reports from the first quarter of 2004 to the first quarter of 2017. Although the vast majority of the reports were submitted from Unites States entities, many reports were submitted to the FDA from all over the world. It was necessary to translate all international and local drug generic and brand names, dates, weights etc. into a single value. A dictionary of drugs was created using online drug databases and all the known brand names of medications were translated into a single value generic form. All capitalizations and identified misspellings of the drugs were recognized and homogenized into a single value.

### Choosing the cohorts

A total of 9,220,343 individual FAERS and AERS reports were collected. 582,889 reports where the patients were treated for pain were selected into a “Pain Cohort” if the “indication” field contained at least one of the following terms: pain, back pain, arthralgia, headache, fibromyalgia, neuralgia, peripheral neuropathy, pain in extremity, pain management, migraine, etc. (see S1 Text for the full list of 112 terms used to define the cohort).

Patients co-treated with known antidepressants in the following classes: SSRIs, SNRIs, serotonin modulators and stimulators (SMSs), tricyclic antidepressants (TCAs), serotonin agonists and reuptake inhibitors (SARIs), norepinephrine reuptake inhibitors (NRIs), tetracyclic antidepressants (TeCAs), monoamine oxidase inhibitors (MAOIs), and DNRIs were excluded from the Pain cohort to avoid confounding factors affecting the frequencies of depression related terms (see the full list of 61 excluded antidepressants and 11 off-label antidepressants in S2 Appendix).

Reports which included unconventional drugs with antidepressant effects such as ketamine, botulinum toxin, and minocycline were also excluded (S2 Appendix) along with reports where the outcome reported was death. The data set was narrowed down to 430,783 reports. The Pain cohort was further split into 2 groups of reports where patients received pain medications that included NSAIDs and pain medication that did not include NSAIDs (S3 Appendix). The NSAID group contained 139,072 records and the non-NSAID group contained 291,711 records.

**Fig 1:**
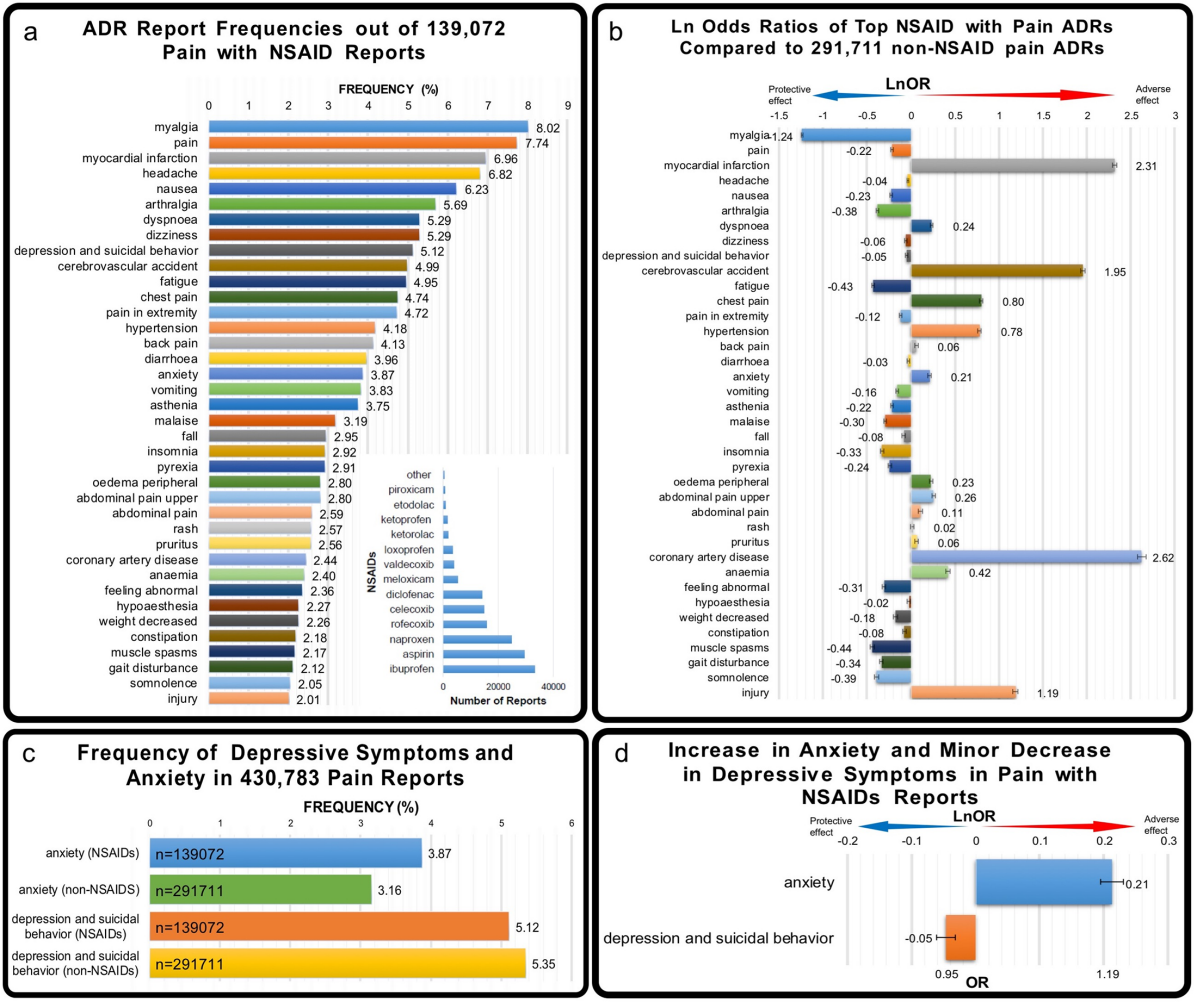
Comparison of ADRs for NSAID class used for pain treatment. (a) Top adverse event frequencies in patients who took NSAIDs for Pain. Adverse events with frequencies above 2% were reported. (b) Ln odds ratios were calculated by comparing patients who took NSAIDs for Pain (n = 139072) and patients who took other medications for Pain (n = 291711). (c) Frequency of anxiety and depression in both cohorts shown. (d) LnOR values for anxiety and depression and suicidal behavior were calculated for NSAID and non-NSAID Pain reports.

Frequencies of all reported adverse effects for both cohorts were calculated and top frequencies were reported (Fig 1). Odds ratios and natural log odds ratios (LnORs, see definition below) for each adverse effect were calculated (Fig 1). Negative values of LnOR with 95% confidence interval not crossing 0 indicates a protective effect of the drug of interest. Conversely, positive values of LnOR with 95% confidence interval not crossing 0 indicates a deleterious effect.

**Fig 2:**
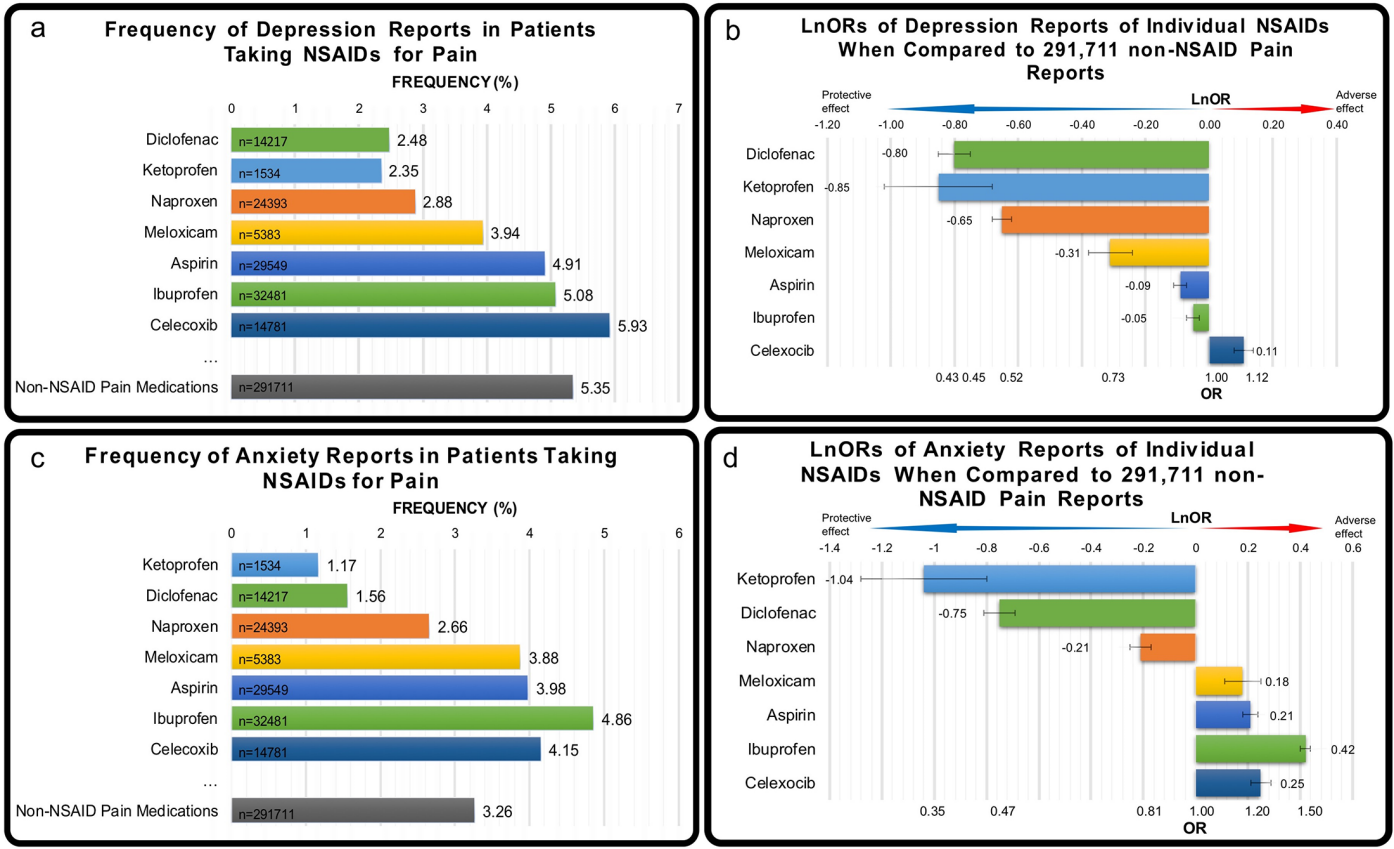
Comparison of ADRs for individual NSAIDs. (a) Adverse event frequencies for depression for each NSAID taken for Pain indications. (b) Logarithm of odds ratios (LnORs) for depression and suicidal behavior were calculated by comparing patients who took the specific NSAID for pain and patients who took non-NSAIDs for Pain. (c) Adverse event frequencies for anxiety for each NSAID taken for Pain indications. (d) LnORs for anxiety were calculated by comparing patients who took the specific NSAID for Pain and patients who took non-NSAIDs for Pain. Negative values of LnOR in (b) and (d) indicate protective effects of ketoprofen, diclofenac and naproxen for both anxiety and depression in patients with Pain.

The NSAID cohort was further divided into patients who received the following NSAIDs for pain and inflammation: ibuprofen n = 32,481, aspirin n = 29,549, naproxen n = 24,393, celecoxib n = 14,781, diclofenac n = 14,217, meloxicam n = 5,383, and ketoprofen = 1,534 (S1 Fig). Each individual NSAIDs’ frequency of anxiety and depression (S4 Appendix) reports was calculated (Fig 2) and LnORs were calculated by comparison with anxiety and depression report frequencies in non-NSAID Pain reports (Fig 2).

### Statistical analysis

#### Descriptive statistics

The most frequent side effects (or Adverse Drug Reactions, ADR) are shown in Figs 1 and 2. Frequency for each side effect was calculated by the equation:

$$\text{Frequency}=\frac{\left(Number\;of\;Records\;with\;ADR\right)}{\left(Number\;of\;Patient\;Records\right)}$$ (1)

#### Comparative statistics

ADR report rates were compared via the Ln Odds Ratio (LnOR) for Figs 1b, 1d, 2b and 2d using the following equations:

$$OR=\;\frac{ad}{bc}$$ (2)

a = Number in exposed group with an adverse event
b = Number in exposed group with *no* adverse event
c = Number in control group with the adverse event
d = Number in control group with *no* adverse event

LnOR was defined and calculated by the following equation:

LnOR=Ln(OR) (3)

Standard Error (SE) of the *LnOR* value was calculated by the following equation:

$$SE_{LnOR}=\;\sqrt{\frac{\;1}{a}+\frac{\;1}{b}+\frac{\;1}{c}+\frac{\;1}{d}}$$ (4)

*variables a, b, c, and d as defined in Eq 2

Error bars were computed using 95% confidence interval. The following formula was used:

$$95\%CI=\left[e^{\left(LnOR-1.96\left(SE_{LnOR}\right)\right)}\;,\;e^{\left(LnOR+1.96\left(SE_{LnOR}\right)\right)}\right]$$ (5)

## Results

Our analysis showed that patients listed in the FAERS database who received NSAIDs in addition to other therapeutics for pain had a minor but significant *decrease* in number of depression and suicidal behavior reports (LnOR -0.05 ±0.015) and a minor but significant *increase* in the number of anxiety reports (LnOR 0.21 ±0.017) (Fig 1). However, patients who received diclofenac in addition to other therapeutics had both depression and anxiety decreased by a much larger value: the number of depression and suicidal behavior reports were decreased by LnOR -0.80 ±0.05 and the number of anxiety reports were decreased by LnOR -0.75 ±0.06. Patients who received ketoprofen in addition to other therapeutics had a decreased number of depression and suicidal behavior reports (LnOR -0.85 ±0.17) and a decreased number of anxiety reports (LnOR -1.04 ±0.24). Patients who received naproxen in addition to other therapeutics had a decreased number of depression and suicidal behavior reports (LnOR -0.65 ±0.03) and a decreased number of anxiety reports (LnOR -0.21 ±0.04). Interestingly, only diclofenac, ketoprofen, and naproxen had a decrease in both anxiety and depression ADRs. Meloxicam, aspirin and ibuprofen patients all had a minor but significant decrease in depression and suicidal behavior (LnORs -0.31 ±0.07, -0.09 ±0.02, and -0.05 ±0.02 respectively) but all had an *increase* in anxiety reports (LnORs 0.18 ±0.07, 0.21 ±0.03, and 0.42 ±0.02 respectively). Celecoxib patients had an increase in both depression and suicidal behavior (LnOR 0.11 ±0.03) and anxiety (LnOR 0.25 ±0.04) (Fig 2).

## Discussion

### Spectrum of NSAID antidepressant and anxiolytic properties

Our results derived from the FAERS and AERS records showed that specific NSAIDs, namely diclofenac, naproxen, and ketoprofen, have marked effects on anxiety and depression. These effects were not NSAID-class-wide but specific to these individual NSAIDs. Although previous studies showed mixed results, this population scale analysis of NSAIDs has revealed the robust antidepressant and anxiolytic effects of specific NSAIDs, and lack of these effects for others in the class for patients treated for pain.

### Bringing AA-NSAIDs into modern psychiatric practice

The unique psychiatric effects of diclofenac, ketoprofen and naproxen warrant new terminology. This family of compounds should be referred to as Anxiolytic Antidepressant Non-steroidal Anti-inflammatory Drugs, or *AA-NSAIDs*.

NSAIDs are rapidly absorbed after oral tablet administration and reach maximum plasma therapeutic concentration (time referred to as t_max_) relatively fast: ketoprofen t_max_ = 0.98 hrs[27], diclofenac t_max_ = 0.89hrs[28], and naproxen t_max_ = 1-2hrs[29, 30]. Ketoprofen freely crosses the blood brain barrier[31], ibuprofen, meloxicam, diclofenac, celecoxib have considerable permeability into CNS (64–76% ±0.04 ratio to diazepam)[32]. Although it is well established that NSAIDs’ onset of action for pain management is rapid and occurs at first administration, the timeline of diclofenac, ketoprofen or naproxen’s effects on anxiety and depression are not known. While it is possible that specific NSAIDs may have acute antidepressant effects, the nature of the FAERS data utilized in this study does not allow us to make definite conclusions about the effect onset. Prospective clinical trials are necessary to establish dosing guidelines for NSAIDs for depression and anxiety due to pain and inflammation.

Furthermore, diclofenac, ketoprofen and naproxen are generic drugs available at most major hospitals and pharmacies globally. The affordability and availability of these agents make their adoption into current psychiatric treatment regiments attractive. These drugs could be evaluated for depression and anxiety in a controlled prospective study of patients with chronic or acute pain and inflammation. Potential risks of NSAIDs as monotherapy or in combination with traditional antidepressants should also be considered. Our findings suggest that NSAIDs have various effects and that some NSAIDs such as aspirin, ibuprofen, and celecoxib could have unfavorable effects on mood and anxiety. Due to the frequent comorbid diagnoses of mood/anxiety and chronic pain disorders, providers may overlook that certain NSAIDs can worsen their patients’ depression and anxiety symptoms. Switching those specific ‘depression-unfavorable’ NSAIDs to ‘depression-favorable’ AA-NSAIDs in these situations may potentially provide additive benefits in improving mood and anxiety symptoms.

Currently, the selection of treatment for psychiatric disorders for a given patient is somewhat variable. There are very few clinical biomarkers that are available or used to guide treatment for individual patients. Guiding psychiatric treatment using inflammatory biomarkers such as IL-6, and IL-8 may lead to selection of better drugs to treat each patient’s specific pathophysiological disruptions that contribute to their depression or anxiety. Monoamine antidepressant therapy may be ineffective in patients whose depression and anxiety are driven by pain and inflammatory processes. Diclofenac, ketoprofen and naproxen’s effects on inflammatory systems in the CNS make these drugs possible candidates for treatment of cases of depression and anxiety deemed inflammatory in nature. Clinical trials will be necessary to establish treatment guidelines to aid clinicians in proper management of pain and inflammation-related depression and anxiety.

## Study limitations

The study represents only a subset of actual cases because of the voluntary nature of FAERS and AERS reporting. For example, a recent study found that FAERS adverse events were significantly underreported for statins[33], Another study found that a lot of reporting to FAERS was overreported and was biased by newsworthiness, legal and scientific influences[34]. Additionally, the absence of comprehensive medical records further dilutes the scope of our analysis, thus our calculated frequencies and odds ratios of adverse events are not true absolute values for the population. Based upon the FDA data source, it is also not possible to discriminate by the severity, duration and nature of depression, anxiety and suicidal behavior symptoms. Since we do not know the duration of depressive symptoms or the duration of antidepressant effects, it is not trivial to convert our results into a specific treatment recommendation. However, 430 thousand reports used for this analysis and choice of control cohorts provided evidence of substantial clinical value with convincingly narrow confidence intervals.

## Supporting information

S1 Appendix Pain and inflammation related indication list. (DOCX)

S2 Appendix Excluded antidepressant list. (DOCX)

S3 Appendix NSAID list. (DOCX)

S4 Appendix Depression, suicidal behavior, and anxiety variants in the adverse event reports. (DOCX)

S5 Appendix Further explanations of the differential psychiatric effects of NSAIDs. (DOCX)

S1 Fig Flowchart of cohort selection. Patients were divided into two cohorts.
(JPG)

S2 Fig NSAID pharmacology. Pharmacology of NSAIDs, ketoprofen, diclofenac, naproxen, meloxicam, aspirin, ibuprofen, and celecoxib at COX, PPAR, and interleukin pathways. The LnOR of depression and suicidal behavior and anxiety is also shown as a heatmap in order to illustrate the clinical outcomes associated with each drug entity (as shown in Fig 2).
(JPG)

S3 Fig Interferon adverse effects. (a) Top adverse event frequencies in patients who took Interferons for hepatitis indication. Adverse events with frequencies above 2.8% were reported. (b) Ln odds ratios were calculated by comparing patients who had interferon treatment for hepatitis (n = 66,640) and patients who took other medications for hepatitis (n = 35,462). (c) Frequency of anxiety and depression in both cohorts shown. (d) Ln odd ratios for anxiety and depression and suicidal behavior were calculated for interferon and non-interferon reports.
(JPG)
