# Supplementary material for: Population scale retrospective analysis reveals distinctive antidepressant and anxiolytic effects of diclofenac, ketoprofen and naproxen in patients with pain
Source: PLoS One. 2018 Apr 18;13(4):e0195521. doi: 10.1371/journal.pone.0195521 (PMC5905979; doi:10.1371/journal.pone.0195521)
Supplement: S1 Appendix — (DOCX) [file pone.0195521.s001.docx]

**S1 Appendix. Pain and inflammation related indication list.**

Pain, acute pain, chronic pain, acute-on-chronic pain, back pain, neck pain, breakthrough pain, pain in extremity, abdominal pain, abdominal pain upper, musculoskeletal pain, pain management, complex regional pain syndrome, gastrointestinal pain, spinal pain, pelvic pain, procedural pain, facial pain, bone pain, pain prophylaxis, oropharyngeal pain, ear pain, bladder pain, eye pain, vulvovaginal pain, inflammatory pain, chronic inflammatory demyelinating polyradiculoneuropathy, small fibre neuropathy, polyneuropathy, sciatic nerve neuropathy, diabetic autonomic neuropathy, polyneuropathy idiopathic progressive, peripheral sensory neuropathy, peripheral motor neuropathy, demyelinating polyneuropathy, neuropathy peripheral, diabetic neuropathy, neuropathy, cancer pain, neuropathic pain, pain in jaw, post-traumatic pain, flank pain, tumour pain, testicular pain, shoulder pain, radicular pain, pharyngolaryngeal pain, oral pain, breast pain, pain nos (not otherwise specified), oesophageal pain, myofascial pain syndrome, cervical root pain, abdominal pain lower, visceral pain, vascular pain, uterine pain, sinus pain, prostatic pain, pleuritic pain, pain of skin, painful respiration, nerve pain, low back pain, labour pain, joint pain, growing pains, migraine, complicated migraine, migraine prophylaxis, migraine with aura, migraine without aura, basilar migraine, hemiplegic migraine, retinal migraine, ophthalmoplegic migraine, migraine type headaches, menstrual migraine, abdominal migraine, status migrainosus, familial hemiplegic migraine, chronic migraine, vestibular migraine, menstrual cramps, cramp, cramps, premenstrual cramps, myalgia, neuralgia, trigeminal neuralgia, occipital neuralgia, post herpetic neuralgia, proctalgia, facial neuralgia, polymyalgia rheumatica, meralgia paraesthetica, rhinalgia, polymyalgia, neuralgia nos, morton's neuralgia, fibromyalgia, arthralgia, cervicogenic headache, vascular headache, post-traumatic headache, tension headaches, chronic headaches, headache, cluster headache, sinus headache, and toothache.
