# Supplementary material for: Population scale retrospective analysis reveals distinctive antidepressant and anxiolytic effects of diclofenac, ketoprofen and naproxen in patients with pain
Source: PLoS One. 2018 Apr 18;13(4):e0195521. doi: 10.1371/journal.pone.0195521 (PMC5905979; doi:10.1371/journal.pone.0195521)
Supplement: S2 Appendix — (DOCX) [file pone.0195521.s002.docx]

**S2 Appendix. Excluded antidepressant list.**

Sertraline, fluoxetine, citalopram, escitalopram, paroxetine, fluvoxamine, venlafaxine, desvenlafaxine, duloxetine, levomilnacipran , amitriptiyline, amoxapine, clomipramine, desipramine, nortriptyline, doxepin, imipramine, protriptyline, trimipramine, maprotiline, bupropion, vilazadone, nefazodone, trazodone, vortioxetine, mirtazapine, isocarboxazid, phenelzine, tranylcypromine, selegiline, minacipran, tofenacin, etoperidone, amitriptylinoxide, dibenzepin, dimetacrine, dosulepin , adapin, lofepramine, melitracen, nitroxazepine, noxiptiline, pipofezine, butriptyline, demexiptiline, imipraminoxide, iprindole, metapramine, propizepine, quinupramine, mianserin, setiptiline, caroxazone, metralindole, moclobemide, pirindole, toloxatone, eprobemide, minaprine.

Atypical/off-label antidepressants:

Minocyline, ketamine, botox, botulinum, botox cosmetic,onabotulinumtoxin A, abobotulinumtoxin A, botulinum toxin type A, rimabotulinumtoxin B, incobotulinumtoxin A, botulinum toxin type B, botox purified neurotoxin complex.
