# Supplementary material for: Population scale retrospective analysis reveals distinctive antidepressant and anxiolytic effects of diclofenac, ketoprofen and naproxen in patients with pain
Source: PLoS One. 2018 Apr 18;13(4):e0195521. doi: 10.1371/journal.pone.0195521 (PMC5905979; doi:10.1371/journal.pone.0195521)
Supplement: S3 Appendix — (DOCX) [file pone.0195521.s003.docx]

**S3 Appendix. NSAID list.**

naproxen, ibuprofen, rofecoxib, diclofenac, tolmetin, sulindac aceclofenac ketorolac, ketoprofen, etodolac, nabumetone, celecoxib, acetylsalicylic acid/aspirin, indomethacin, piroxicam, oxaprozin, salsalate, diflunisal, ampiroxicam, tenoxicam, droxicam, lornoxicam, chlortenoxicam, meloxicam, isoxicam, dexibuprofen, fenoprofen, dexketoprofen, flurbiprofen, loxoprofen, mefanemic acid, phenylbutazone, meclofenamic acid, flufenamic acid, tolfenamic acid, valdecoxib, parecoxib, lumiracoxib, etoricoxib, firocoxib.
