# Supplementary material for: Population scale retrospective analysis reveals distinctive antidepressant and anxiolytic effects of diclofenac, ketoprofen and naproxen in patients with pain
Source: PLoS One. 2018 Apr 18;13(4):e0195521. doi: 10.1371/journal.pone.0195521 (PMC5905979; doi:10.1371/journal.pone.0195521)
Supplement: S4 Appendix — (DOCX) [file pone.0195521.s004.docx]

**S4 Appendix**. **Depression, suicidal behavior, and anxiety variants in the adverse event reports.**

*Depression and suicidal behavior variants in adverse event reports:*

Depression, depressed mood, major depression, adjustment disorder with depressed mood, depressive symptom, adjustment disorder with mixed anxiety and depression, agitated depression, persistent depressive disorder, depression suicidal, adjustment disorder with anxiety and depressed mood, suicidal ideation, suicide attempt, suicidal behaviour.

*Anxiety variants in adverse event reports:*

Anxiety, anxiety disorder, generalized anxiety disorder, adjustment disorder with anxiety and depressed mood, social anxiety disorder, adjustment disorder with anxiety.
