# Supplementary material for: Population scale retrospective analysis reveals distinctive antidepressant and anxiolytic effects of diclofenac, ketoprofen and naproxen in patients with pain
Source: PLoS One. 2018 Apr 18;13(4):e0195521. doi: 10.1371/journal.pone.0195521 (PMC5905979; doi:10.1371/journal.pone.0195521)
Supplement: S5 Appendix — (DOCX) [file pone.0195521.s005.docx]

**S5 Appendix**. **Further explanations of the differential psychiatric effects of NSAIDs.**

S2 Fig summarizes the complex pharmacology of NSAIDs at COX, PPAR, and interleukin pathways. Not surprisingly, all NSAIDs inhibit COX enzymes, however not all elements of their unique pharmacological fingerprints are conserved class wide (S2 Fig). When comparing clinical outcomes of each NSAID with their pharmacology, it becomes apparent that inhibition of COX enzymes does *not* necessarily lead to mitigation of depression and anxiety (S2 Fig). Logically, other pathways differently co-affected by NSAIDs may be important for this effect

*PPAR-gamma activity doesn’t explain differential psychiatric effects of NSAIDs*

We investigated the potential of PPAR-gamma modulation to discriminate between psychiatric effects of NSAIDs. Due to the previously observed antidepressant effect of pioglitazone[1], a PPAR-gamma activation was a discriminating feature candidate. However, as evident from Supplement Figure 2, the PPAR-gamma activity of NSAIDs did not correlate with the clinical outcomes in FAERS we observed. Specifically, ketoprofen was observed to have the largest effect on depression and anxiety, while it had no direct PPAR-gamma activity[2].

*Modulation of cytokine signaling may explain NSAID mitigation of depression*

Previous studies have revealed that therapeutic administration of interferon-alpha, which induces IL-6 and to some extent IL-1, tumor necrosis factor (TNF)-alpha, and IL-8 production, leads to depression in nearly half of patients treated for viral infections and other conditions[3,4]. We confirmed this trend by analyzing over sixty thousand FAERS patients treated with interferons for Hepatitis A, B, and C, and compared them to patients who were treated with non-interferon medications. There was a significant increase in both depression and anxiety in patients who received interferon-alpha, further supporting the theory of cytokine induced depression (Appendix F S. Fig.3 ). When compared to non-depressed individuals, some but not all depressed individuals, have elevated levels of inflammatory cytokines. In these patients increased plasma and cerebrospinal fluid (CSF) concentrations of interleukin IL-6[5,6,7], IL-1, TNF-alpha, C-reactive proteins[8], and IL-8[9] were found. It was also shown that the expression of IL-8 receptor CXCR1 is widely expressed in CNS on glutaminergic, GABA-ergic, and cholinergic neurons. CXCR1 plays a major role in neuronal development and survival, neuronal excitability and neuroimmune response modulation[10].

Direct modulation of chemokine receptor CXCR1 by only certain NSAIDs may be a missing mechanistic link needed to explain their antidepressant and anxiolytic effects. In fact, it has been already established that diclofenac, ketoprofen and naproxen cause inhibition or downregulation of CXCR1[11,12]. Although most NSAIDs decrease IL-6 and IL-8, only diclofenac, ketoprofen and naproxen have been shown to antagonize CXCR1 (S. Fig 2). This pharmacology may explain the previous results shown, that diclofenac, ketoprofen and naproxen have unique antidepressant effects. CXCR1 may be an important new target for new psychiatric therapeutics.

Supplementary information for S2 Fig.

|  | COX-1  IC50 μM | COX-2  IC50 μM | PPAR−γ | IL-6 levels | IL-8 levels | CXCR1 |
| --- | --- | --- | --- | --- | --- | --- |
| Ketoprofen | **0.047[13]** | 0.24[13] | No significant effect[2] | No effect  At 100 μM[20] | Reduced production[30] | **Inhibition[11]** |
| Diclofenac | 0.076[14] | **0.026[14]** | Partial agonist, dose dependent  Ki=700nM  Activity=25 μM[16]  EC50=1.69 μM[17] | Inhibits production[21] | No effect on production[21] | **Inhibition**  **12nM[12]** |
| Naproxen | 2.2[15] | **1.3[15]** | Activates[18] | Decreased production[24] | Reduce Il-8/neutrophil migration by  blocked the PI3K/Akt pathway[25] | **Inhibition 35%[11]** |
| Meloxicam | 37.0[14] | **6.1[14]** | Upregulated PPAR expression[22] | **Increased production[26,27]** | Unaffected by therapeutic concentration[26] |  |
| Aspirin | **1.67[15]** | 278[15] | Upregulated PPAR expression[23] | Decreased levels[28] | Decreased expression[31] |  |
| Ibuprofen | **12[14]** | 80[14] | Partial agonist, dose dependent  EC50 =56.8 μM[17]  Activates[18] | Decreased production[29] | Reduce Il-8/neutrophil migration  By integrin downregulation[25] |  |
| Celecoxib | 82[14] | **6.8[14]** | Activates PPARγ/PTEN  signaling[19] | Decreased production[29] | **No effect on Il-8 levels[32]** |  |

References cited for S2 Fig.

1. Colle R, de Larminat D, Rotenberg S, Hozer F, Hardy P, Verstuyft C, et al. Pioglitazone could induce remission in major depression: a meta-analysis. Neuropsychiatr Dis Treat. 2017;13:9-16. Epub 2016/12/19. doi: 10.2147/NDT.S121149. PubMed PMID: 28031713; PubMed Central PMCID: PMCPMC5182046.

2. Yamazaki R, Kusunoki N, Matsuzaki T, Hashimoto S, Kawai S. Nonsteroidal anti-inflammatory drugs induce apoptosis in association with activation of peroxisome proliferator-activated receptor gamma in rheumatoid synovial cells. J Pharmacol Exp Ther. 2002;302(1):18-25. PubMed PMID: 12065695.

3. Bonaccorso S, Marino V, Biondi M, Grimaldi F, Ippoliti F, Maes M. Depression induced by treatment with interferon-alpha in patients affected by hepatitis C virus. J Affect Disord. 2002;72(3):237-41. PubMed PMID: 12450640.

4. Murakami Y, Ishibashi T, Tomita E, Imamura Y, Tashiro T, Watcharanurak K, et al. Depressive symptoms as a side effect of Interferon-α therapy induced by induction of indoleamine 2,3-dioxygenase 1. Sci Rep. 2016;6:29920. Epub 2016/07/20. doi: 10.1038/srep29920. PubMed PMID: 27436416; PubMed Central PMCID: PMCPMC4951771.

5. Raison CL, Capuron L, Miller AH. Cytokines sing the blues: inflammation and the pathogenesis of depression. Trends Immunol. 2006;27(1):24-31. Epub 2005/11/28. doi: 10.1016/j.it.2005.11.006. PubMed PMID: 16316783; PubMed Central PMCID: PMCPMC3392963.

6. Alesci S, Martinez PE, Kelkar S, Ilias I, Ronsaville DS, Listwak SJ, et al. Major depression is associated with significant diurnal elevations in plasma interleukin-6 levels, a shift of its circadian rhythm, and loss of physiological complexity in its secretion: clinical implications. J Clin Endocrinol Metab. 2005;90(5):2522-30. Epub 2005/02/10. doi: 10.1210/jc.2004-1667. PubMed PMID: 15705924.

7. Musselman DL, Miller AH, Porter MR, Manatunga A, Gao F, Penna S, et al. Higher than normal plasma interleukin-6 concentrations in cancer patients with depression: preliminary findings. Am J Psychiatry. 2001;158(8):1252-7. doi: 10.1176/appi.ajp.158.8.1252. PubMed PMID: 11481159.

8. Dowlati Y, Herrmann N, Swardfager W, Liu H, Sham L, Reim EK, et al. A meta-analysis of cytokines in major depression. Biol Psychiatry. 2010;67(5):446-57. Epub 2009/12/16. doi: 10.1016/j.biopsych.2009.09.033. PubMed PMID: 20015486.

9. Oliveira Miranda D, Soares de Lima TA, Ribeiro Azevedo L, Feres O, Ribeiro da Rocha JJ, Pereira-da-Silva G. Proinflammatory cytokines correlate with depression and anxiety in colorectal cancer patients. Biomed Res Int. 2014;2014:739650. Epub 2014/09/17. doi: 10.1155/2014/739650. PubMed PMID: 25309921; PubMed Central PMCID: PMCPMC4182686.

10. Danik M, Puma C, Quirion R, Williams S. Widely expressed transcripts for chemokine receptor CXCR1 in identified glutamatergic, gamma-aminobutyric acidergic, and cholinergic neurons and astrocytes of the rat brain: a single-cell reverse transcription-multiplex polymerase chain reaction study. J Neurosci Res. 2003;74(2):286-95. doi: 10.1002/jnr.10744. PubMed PMID: 14515358.

11. Allegretti M, Bertini R, Cesta MC, Bizzarri C, Di Bitondo R, Di Cioccio V, et al. 2-Arylpropionic CXC chemokine receptor 1 (CXCR1) ligands as novel noncompetitive CXCL8 inhibitors. J Med Chem. 2005;48(13):4312-31. doi: 10.1021/jm049082i. PubMed PMID: 15974585.

12. Sablone MR, Cesta MC, Moriconi A, Aramini A, Bizzarri C, Di Giacinto C, et al. Structure-Activity Relationship of novel phenylacetic CXCR1 inhibitors. Bioorg Med Chem Lett. 2009;19(15):4026-30. Epub 2009/06/13. doi: 10.1016/j.bmcl.2009.06.027. PubMed PMID: 19560921.

13. Warner TD, Giuliano F, Vojnovic I, Bukasa A, Mitchell JA, Vane JR. Nonsteroid drug selectivities for cyclo-oxygenase-1 rather than cyclo-oxygenase-2 are associated with human gastrointestinal toxicity: a full in vitro analysis. Proc Natl Acad Sci U S A. 1999;96(13):7563-8. PubMed PMID: 10377455; PubMed Central PMCID: PMCPMC22126.

14. Kato M, Nishida S, Kitasato H, Sakata N, Kawai S. Cyclooxygenase-1 and cyclooxygenase-2 selectivity of non-steroidal anti-inflammatory drugs: investigation using human peripheral monocytes. J Pharm Pharmacol. 2001;53(12):1679-85. PubMed PMID: 11804398.

15. Mitchell JA, Akarasereenont P, Thiemermann C, Flower RJ, Vane JR. Selectivity of nonsteroidal antiinflammatory drugs as inhibitors of constitutive and inducible cyclooxygenase. Proc Natl Acad Sci U S A. 1993;90(24):11693-7. PubMed PMID: 8265610; PubMed Central PMCID: PMCPMC48050.

16. Adamson DJ, Frew D, Tatoud R, Wolf CR, Palmer CN. Diclofenac antagonizes peroxisome proliferator-activated receptor-gamma signaling. Mol Pharmacol. 2002;61(1):7-12. PubMed PMID: 11752200.

17. Puhl AC, Milton FA, Cvoro A, Sieglaff DH, Campos JC, Bernardes A, et al. Mechanisms of peroxisome proliferator activated receptor γ regulation by non-steroidal anti-inflammatory drugs. Nucl Recept Signal. 2015;13:e004. Epub 2015/10/05. doi: 10.1621/nrs.13004. PubMed PMID: 26445566; PubMed Central PMCID: PMCPMC4594550.

18. Jaradat MS, Wongsud B, Phornchirasilp S, Rangwala SM, Shams G, Sutton M, et al. Activation of peroxisome proliferator-activated receptor isoforms and inhibition of prostaglandin H(2) synthases by ibuprofen, naproxen, and indomethacin. Biochem Pharmacol. 2001;62(12):1587-95. PubMed PMID: 11755111.

19. Chu TH, Chan HH, Kuo HM, Liu LF, Hu TH, Sun CK, et al. Celecoxib suppresses hepatoma stemness and progression by up-regulating PTEN. Oncotarget. 2014;5(6):1475-90. doi: 10.18632/oncotarget.1745. PubMed PMID: 24721996; PubMed Central PMCID: PMCPMC4039225.

20. Kang BS, Chung EY, Yun YP, Lee MK, Lee YR, Lee KS, et al. Inhibitory effects of anti-inflammatory drugs on interleukin-6 bioactivity. Biol Pharm Bull. 2001;24(6):701-3. PubMed PMID: 11411563.

21. Henrotin YE, Labasse AH, Simonis PE, Zheng SX, Deby GP, Famaey JP, et al. Effects of nimesulide and sodium diclofenac on interleukin-6, interleukin-8, proteoglycans and prostaglandin E2 production by human articular chondrocytes in vitro. Clin Exp Rheumatol. 1999;17(2):151-60. PubMed PMID: 10342040.

22. Xin B, Yokoyama Y, Shigeto T, Futagami M, Mizunuma H. Inhibitory effect of meloxicam, a selective cyclooxygenase-2 inhibitor, and ciglitazone, a peroxisome proliferator-activated receptor gamma ligand, on the growth of human ovarian cancers. Cancer. 2007;110(4):791-800. doi: 10.1002/cncr.22854. PubMed PMID: 17582802.

23. Yiqin Y, Meilin X, Jie X, Keping Z. Aspirin inhibits MMP-2 and MMP-9 expression and activity through PPARalpha/gamma and TIMP-1-mediated mechanisms in cultured mouse celiac macrophages. Inflammation. 2009;32(4):233-41. doi: 10.1007/s10753-009-9125-3. PubMed PMID: 19462226.

24. Martínez Cairo S, Salgado Legorreta C, Martínez Zurita F. [Effect of naproxen on serum concentrations of IL-I, IL-6, and TNF in patients with osteoarthritis]. Rev Alerg Mex. 2001;48(4):119-22. PubMed PMID: 11593916.

25. Bertolotto M, Contini P, Ottonello L, Pende A, Dallegri F, Montecucco F. Neutrophil migration towards C5a and CXCL8 is prevented by non-steroidal anti-inflammatory drugs via inhibition of different pathways. Br J Pharmacol. 2014;171(14):3376-93. doi: 10.1111/bph.12670. PubMed PMID: 24597536; PubMed Central PMCID: PMCPMC4105927.

26. Rainsford KD, Ying C, Smith FC. Effects of meloxicam, compared with other NSAIDs, on cartilage proteoglycan metabolism, synovial prostaglandin E2, and production of interleukins 1, 6 and 8, in human and porcine explants in organ culture. J Pharm Pharmacol. 1997;49(10):991-8. PubMed PMID: 9364409.

27. Scheller J, Chalaris A, Schmidt-Arras D, Rose-John S. The pro- and anti-inflammatory properties of the cytokine interleukin-6. Biochim Biophys Acta. 2011;1813(5):878-88. Epub 2011/02/04. doi: 10.1016/j.bbamcr.2011.01.034. PubMed PMID: 21296109.

28. Ikonomidis I, Andreotti F, Economou E, Stefanadis C, Toutouzas P, Nihoyannopoulos P. Increased proinflammatory cytokines in patients with chronic stable angina and their reduction by aspirin. Circulation. 1999;100(8):793-8. PubMed PMID: 10458713.

29. Gallelli L, Galasso O, Falcone D, Southworth S, Greco M, Ventura V, et al. The effects of nonsteroidal anti-inflammatory drugs on clinical outcomes, synovial fluid cytokine concentration and signal transduction pathways in knee osteoarthritis. A randomized open label trial. Osteoarthritis Cartilage. 2013;21(9):1400-8. doi: 10.1016/j.joca.2013.06.026. PubMed PMID: 23973155.

30. Wang LM, Toyoshima A, Mineshita S, Wang XX, Yamamoto T, Nomura Y, et al. The anti-inflammatory effects of ketoprofen in animal experiments. Drugs Exp Clin Res. 1997;23(1):1-6. PubMed PMID: 9093816.

31. Yang YY, Hu CJ, Chang SM, Tai TY, Leu SJ. Aspirin inhibits monocyte chemoattractant protein-1 and interleukin-8 expression in TNF-alpha stimulated human umbilical vein endothelial cells. Atherosclerosis. 2004;174(2):207-13. doi: 10.1016/j.atherosclerosis.2004.01.024. PubMed PMID: 15136050.

32. Bianchi M, Broggini M, Balzarini P, Franchi S, Sacerdote P. Effects of nimesulide on pain and on synovial fluid concentrations of substance P, interleukin-6 and interleukin-8 in patients with knee osteoarthritis: comparison with celecoxib. Int J Clin Pract. 2007;61(8):1270-7. Epub 2007/06/22. doi: 10.1111/j.1742-1241.2007.01453.x. PubMed PMID: 17590218.
