# Supplementary figures and images for: Population scale retrospective analysis reveals distinctive antidepressant and anxiolytic effects of diclofenac, ketoprofen and naproxen in patients with pain
Source: PLoS One. 2018 Apr 18;13(4):e0195521. doi: 10.1371/journal.pone.0195521 (PMC5905979; doi:10.1371/journal.pone.0195521)

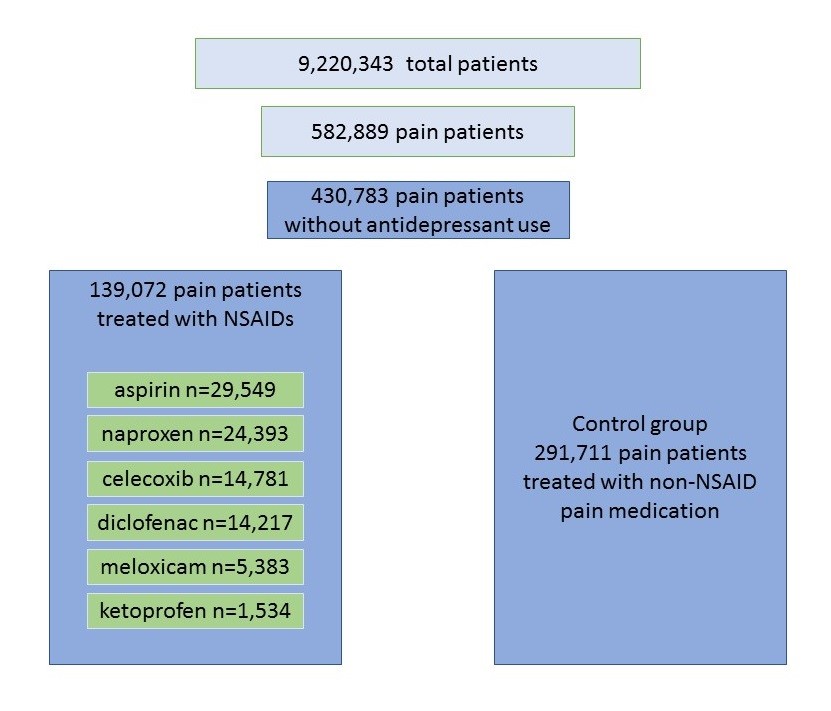

Supplement: S1 Fig — Patients were divided into two cohorts. (JPG) [file pone.0195521.s006.jpg]

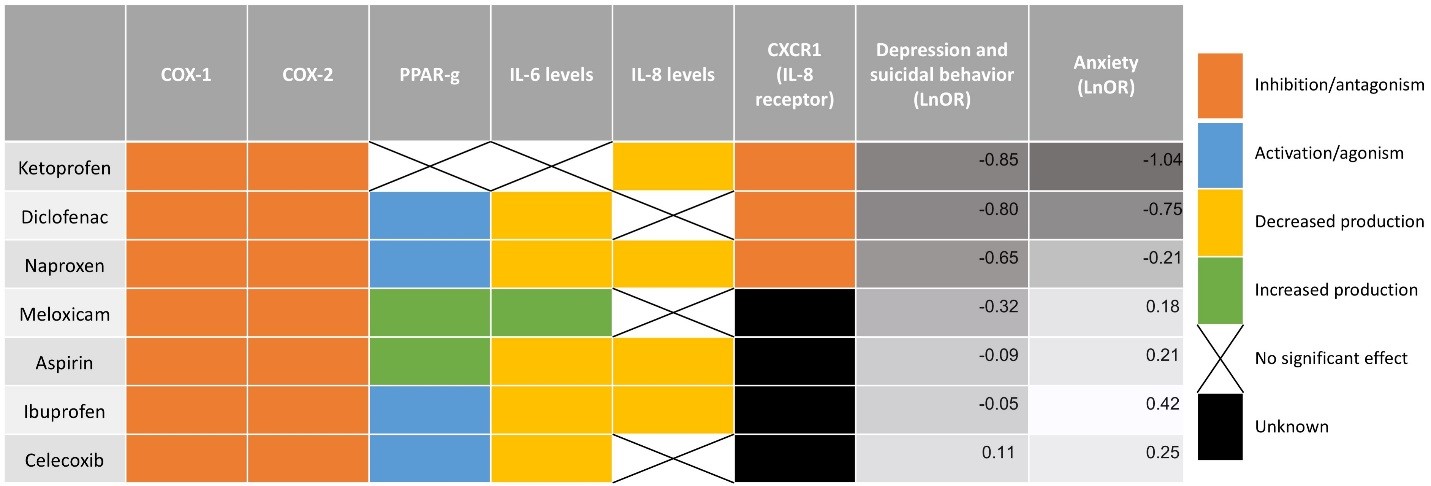

Supplement: S2 Fig — Pharmacology of NSAIDs, ketoprofen, diclofenac, naproxen, meloxicam, aspirin, ibuprofen, and celecoxib at COX, PPAR, and interleukin pathways. The LnOR of depression and suicidal behavior and anxiety is also shown as a heatmap in order to illustrate the clinical outcomes associated with each drug entity (as shown in Fig 2). (JPG) [file pone.0195521.s007.jpg]

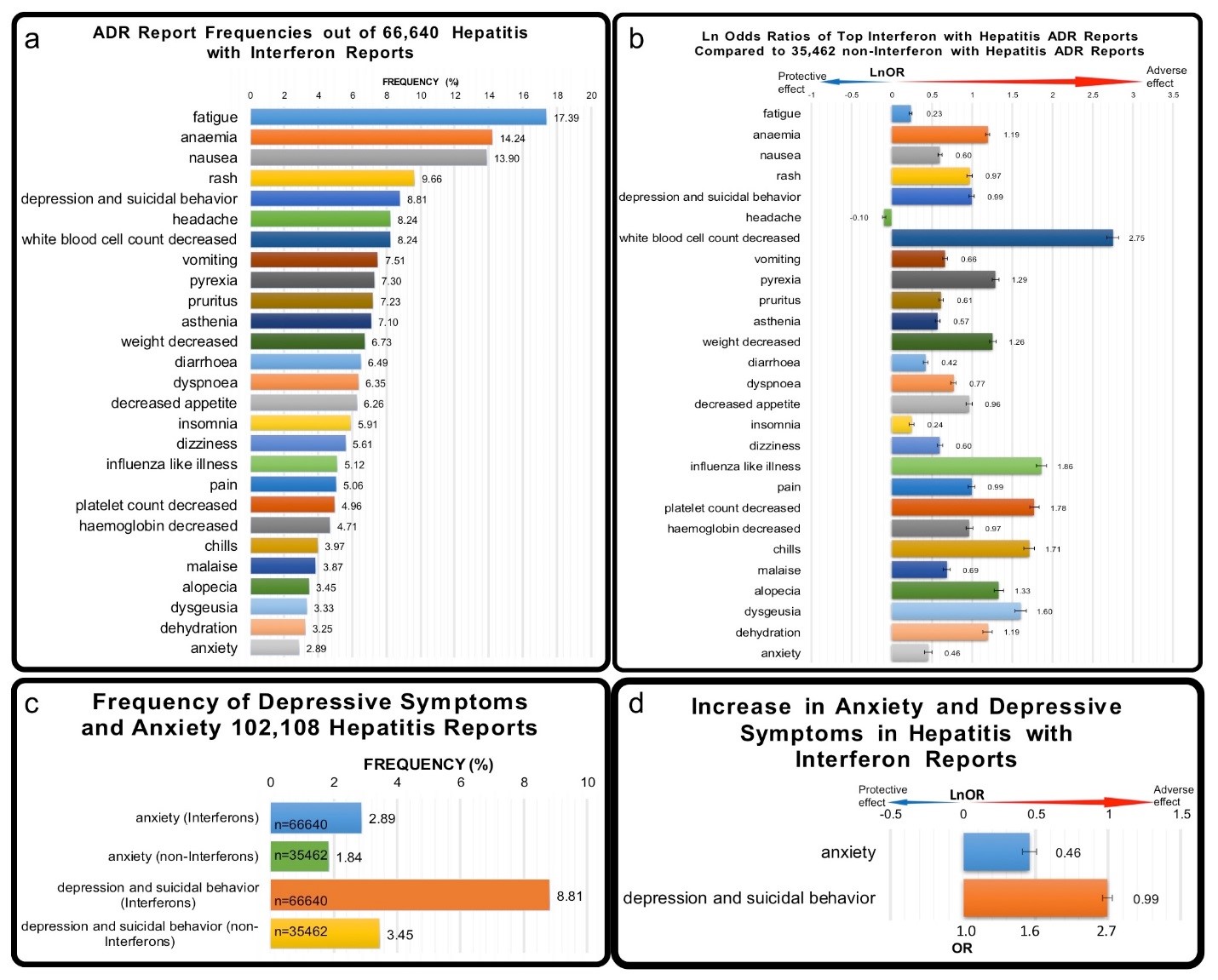

Supplement: S3 Fig — (a) Top adverse event frequencies in patients who took Interferons for hepatitis indication. Adverse events with frequencies above 2.8% were reported. (b) Ln odds ratios were calculated by comparing patients who had interferon treatment for hepatitis (n = 66,640) and patients who took other medications for hepatitis (n = 35,462). (c) Frequency of anxiety and depression in both cohorts shown. (d) Ln odd ratios for anxiety and depression and suicidal behavior were calculated for interferon and non-interferon reports. (JPG) [file pone.0195521.s008.jpg]
